# Non-invasive brain stimulation modulates GABAergic activity in neurofibromatosis 1

**DOI:** 10.1038/s41598-022-21907-9

**Published:** 2022-10-31

**Authors:** Shruti Garg, Steve Williams, JeYoung Jung, Gorana Pobric, Tulika Nandi, Ben Lim, Grace Vassallo, Jonathan Green, D. Gareth Evans, Charlotte J. Stagg, Laura M. Parkes, Stavros Stivaros

**Affiliations:** 1 Division of Neuroscience and Experimental Psychology, School of Biological Sciences, Faculty of Biology, Medicine and Health, University of Manchester, Manchester, UK; 2 Child and Adolescent Mental Health Services, Royal Manchester Children’s Hospital, Central Manchester University Hospitals NHS Foundation Trust, Manchester Academic Health Sciences Centre, Manchester, UK; 3 Division of Informatics, Imaging and Data Sciences, School of Health Sciences, University of Manchester, Manchester, UK; 4 School of Psychology, Precision Imaging Beacon, University of Nottingham, Nottingham, UK; 5 Wellcome Centre for Integrative Neuroimaging, Nuffield Department of Clinical Neurosciences, University of Oxford, Oxford, UK; 6 Manchester Centre for Genomic Medicine, Manchester University NHS Foundation Trust, Manchester, UK; 7 Division of Evolution and Genomic Sciences, Faculty of Biology, Medicine and Health, University of Manchester, Manchester, UK; 8 Geoffrey Jefferson Brain Research Centre, Manchester Academic Health Science Centre, Manchester, UK; 9 Academic Unit of Paediatric Radiology, Royal Manchester Children’s Hospital, Manchester University NHS Foundation Trust, Manchester, UK

**Keywords:** Working memory, Autism spectrum disorders, Development, Neurodevelopmental disorders

## Abstract

Neurofibromatosis 1 (NF1) is a single-gene disorder associated with cognitive phenotypes common to neurodevelopmental conditions such as autism. GABAergic dysregulation underlies working memory impairments seen in NF1. This mechanistic experimental study investigates whether application of anodal transcranial direct current stimulation (atDCS) can modulate GABA and working memory in NF1. Thirty-one NF1 adolescents 11–18 years, were recruited to this single-blind sham-controlled cross-over randomized trial. AtDCS or sham stimulation was applied to the left Dorsolateral Prefrontal Cortex (DLPFC) and MR Spectroscopy was collected before and after intervention in the left DLPFC and occipital cortex. Task-related functional MRI was collected before, during, and after stimulation. Higher baseline GABA+ in the left DLPFC was associated with faster response times on baseline working memory measures. AtDCS was seen to significantly reduced GABA+ and increase brain activation in the left DLPFC as compared to sham stimulation. Task performance was worse in the aTDCS group during stimulation but no group differences in behavioural outcomes were observed at the end of stimulation. Although our study suggests aTDCS modulates inhibitory activity in the DLPFC, further work is needed to determine whether repeated sessions of atDCS and strategies such as alternating current stimulation offer a better therapeutic approach.

## Introduction

Perturbations in gamma-aminobutyric acid (GABA) inhibitory neurotransmission have been postulated to underlie common psychiatric disorders such as schizophrenia and neurodevelopmental conditions such as Autism Spectrum Disorders (ASD)^1,2^. GABAergic neurotransmission plays a central role in homeostatic plasticity mechanisms to maintain a fine balance between excitation/inhibition (E/I) and promote network stability^3^. Indeed, based on findings of preclinical studies, numerous pharmacological therapies (for instance bumetanide^4^, memantine^5^, arbaclofen^6^) hypothesized to target GABAergic neurotransmission have been tried in neuropsychiatric conditions, with the goal of restoring inhibitory/excitatory balance, particularly in the prefrontal cortex. However, human clinical trials of GABAergic drugs have shown limited therapeutic success, highlighting both the fundamental gap in mechanistic translation and need for objective clinical measures linked to the known mechanism of disease^7^. Mechanistic studies in humans are the next important step to fully understand the role of GABAergic signaling and for the development of biologically-targeted therapies for neuropsychiatric disorders.

Syndromic versions of common mental disorders, although rare, can be used to provide important clues about underlying molecular pathogenesis. An example of this is Neurofibromatosis 1 (NF1), a rare single-gene disorder with birth incidence of 1:2700^8^, that is known to be associated with GABAergic dysfunction. Although NF1 is commonly recognised for its cutaneous manifestations and increased tumour predisposition^9^, significant morbidity is caused by cognitive, social and behavioural difficulties^10^. The cognitive-behavioural phenotype in NF1 has been well-described and the disorder presents with many phenotypes seen in common mental illnesses. For instance, up to 70% of individuals with NF1 have impairments in working memory, 25% may have comorbid Autism Spectrum Disorder (ASD) and up to 50% Attention Deficit Hyperactivity Disorder (ADHD)^11,12^. As a monogenic disorder, the underlying neurobiology of NF1 is well-understood through the use of animal models. Mutation of the *Nf1* gene causes disinhibition of the RasMAPK and other downstream signalling pathways resulting in changes of synaptic proteins causing GABAergic overactivity and impairments in synaptic plasticity^13^. More specifically, animal studies have demonstrated that GABAergic dysfunction underlies working memory impairments in NF1 by disrupting corticostriatal activity^14^. Studies of GABA function in vivo are limited, with only two previous reports suggesting GABA dysfunction in children and adults with NF1^15,16^ associated with impairments in cognition, motor skills^17^ and impulse control^18^.

More recently, interventions such as Non-Invasive Brain Stimulation (NIBS) that can be used to modulate cortical plasticity have generated much interest as putative therapeutic treatments for learning impairments such as working memory deficits^19,20^. In this context, anodal transcranial Direct Current Stimulation (atDCS), which involves passing a small electric current through the scalp via scalp electrodes, has been of particular interest. AtDCS has facilitatory effects on the underlying neural tissue and has been shown, using Magnetic Resonance Spectroscopy (MRS), to reduce GABA in the stimulated cortex in healthy populations. This represents a putative mechanism to explain its known local ability both to induce temporary long-term potentiation like effects and to increase network-level functional connectivity^21^. NIBS techniques therefore provide a distinct advantage over pharmacological agents in being able to induce local cortical changes.

In this study, we use NF1 as a model to study how inter-individual differences in GABA function relate to working memory performance—a phenotype shared across several neurodevelopmental disorders. We test the responsiveness of the GABAergic system to atDCS applied to the left dorsolateral prefrontal cortex (DLPFC), a region chosen for its critical role in working memory in humans^22^. Further, functional MRI and EEG studies in NF1 have shown hypoactivation of the left DLPFC^23,24^. We hypothesized that (i) application of atDCS to the left dorsolateral prefrontal cortex (DLPFC) would reduce GABA and improve performance on working memory tasks and (ii) application of aTDCS would increase brain activation in the targeted DLPFC.

## Results

Thirty-one participants participated in the experimental sessions over two separate days. Twenty-nine participants completed both study visits, and two participants only completed one visit (further visits suspended due to COVID-19 related lockdown). The clinical characteristics of the sample are described in Table 1.

**Table 1:** Clinical characteristics of the study sample.

**Males**	15/31
**Age (mean)**	14.7 years (11.4–18.3)
**Pre-existing diagnoses (n)**	
ADHD	8
ASD	3
**Medications**	
Stimulants	6
Atomoxetine	1
**Vineland adaptive behavior composite (mean)**	68.4 (13.0)
**Conners (mean)**	
Inattention T score	78.7 (13.0)
Hyperactivity T score	69.1 (18.2)

### Higher baseline DLPFC GABA+ associated with shorter response times

**Figure 1:**
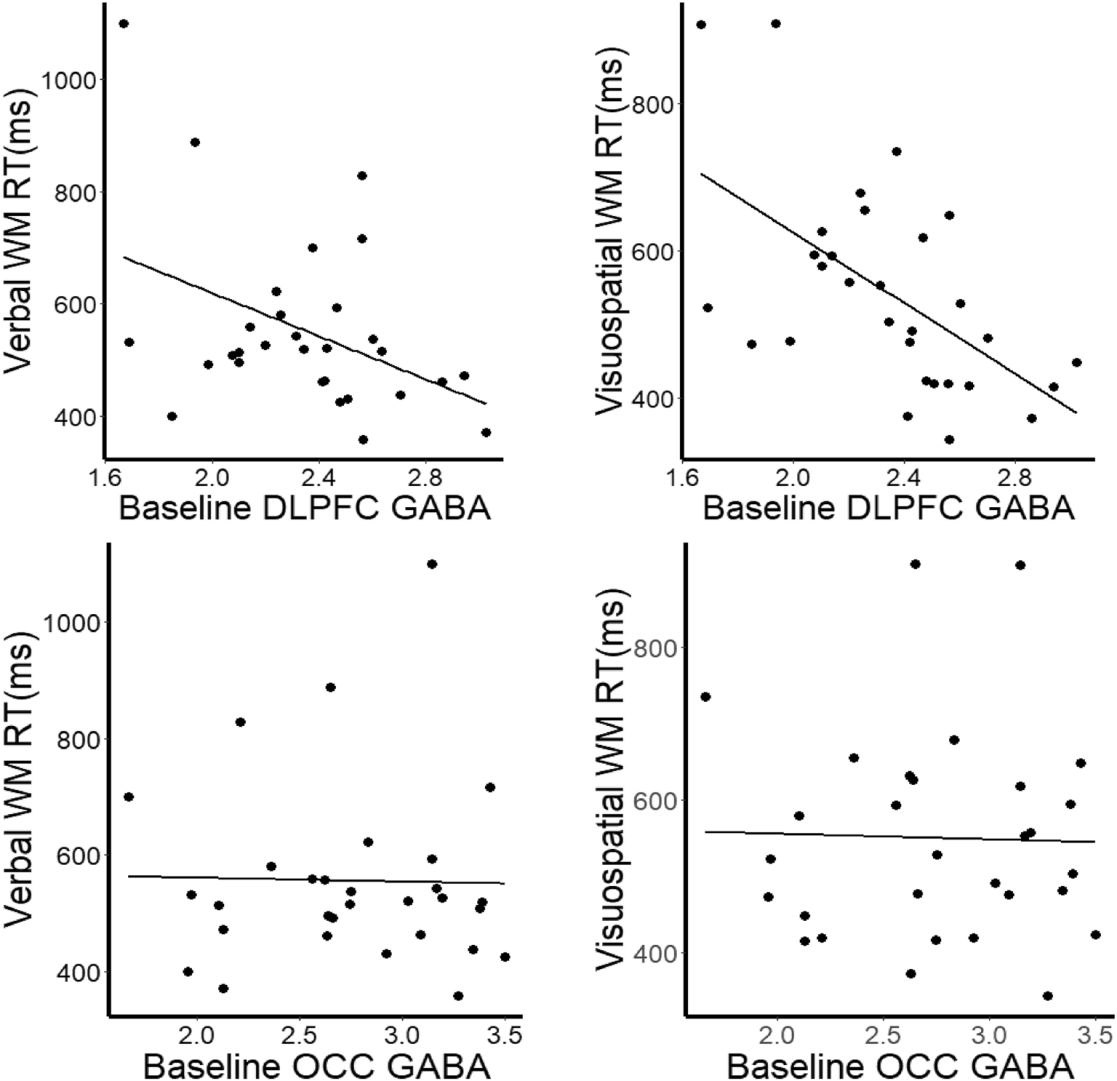
Correlation between verbal and visuospatial working memory response time (RT) with baseline dorsolateral prefrontal cortex (DLPFC) GABA and occipital (OCC). GABA corrected for voxel tissue fraction.

We first tested the relationship between baseline GABA+ (where GABA+ indicates a measurement of GABA and co-edited macromolecules) measured at the first visit to working memory performance. No significant correlation between baseline GABA+ and working memory accuracy was observed (Verbal working memory *r*(27) = 0.28, *p* = 0.13, Visuospatial working memory *r*(27) = 0.13, *p* = 0.49) but there was a significant correlation between GABA+ and RTs on both verbal and visuospatial working memory, such that patients with higher GABA+ showed faster RTs (Verbal working memory *r*(27) = − 0.41 *p* = 0.03, Visuospatial working memory *r*(27) = − 0.58, *p* = 0.001). Higher GABA+ was associated with a lower Inverse Efficiency Score (IES) (Verbal working memory *r*(27) = − 0.44, *p* = 0.02, Visuospatial working memory *r*(27) = − 0.59, *p* = 0.001, Fig. 1). This result was neurotransmitter specific: no significant relationship was observed between Glx and RT (Verbal RT *r*(27) = − 0.08, *p* = 0.69, Visuospatial RT *r*(27) = 0.13, *p* = 0.51) and there was a significant difference between the GABA/Glx correlation coefficients for working memory RT(verbal RT z = − 1.88, *p* = 0.03, visuospatial RT z = − 2.87, p = 0.002). This finding was also anatomically specific: there was no relationship between OCC GABA+ and RT (Verbal RT *r*(27) = − 0.02, *p* = 0.91, Visuospatial RT *r*(27) = − 0.03, *p* = 0.89) and there was a significant difference between the DLPFC/OCC GABA+ and Visuospatial RT correlation coefficients (verbal RT z = − 1.52, *p* = 0.06, Visuospatial RT z = − 2.31, *p* = 0.01).

There was no relationship between parent rated measures and DLPFC GABA+ including baseline adaptive functioning (*r*(27) = 0.08, *p* = 0.66), inattention (*r*(27) = − 0.05, *p* = 0.79) or hyperactivity (*r*(27) = − 0.22, *p* = 0.23). Similarly, no relationship was observed between DLPFC Glx and baseline adaptive function (*r*(27) = − 0.12, *p* = 0.54), inattention (*r*(27) = − 0.09, p = 0.66) or hyperactivity (*r*(27) = − 0.17, *p* = 0.37). No relationship was observed between any of the neurotransmitters in the occipital cortex and parent reported metrics.

### AtDCS is associated with greater reduction in GABA+ relative to sham but there is no effect on behavioural outcomes

**Figure 2:**
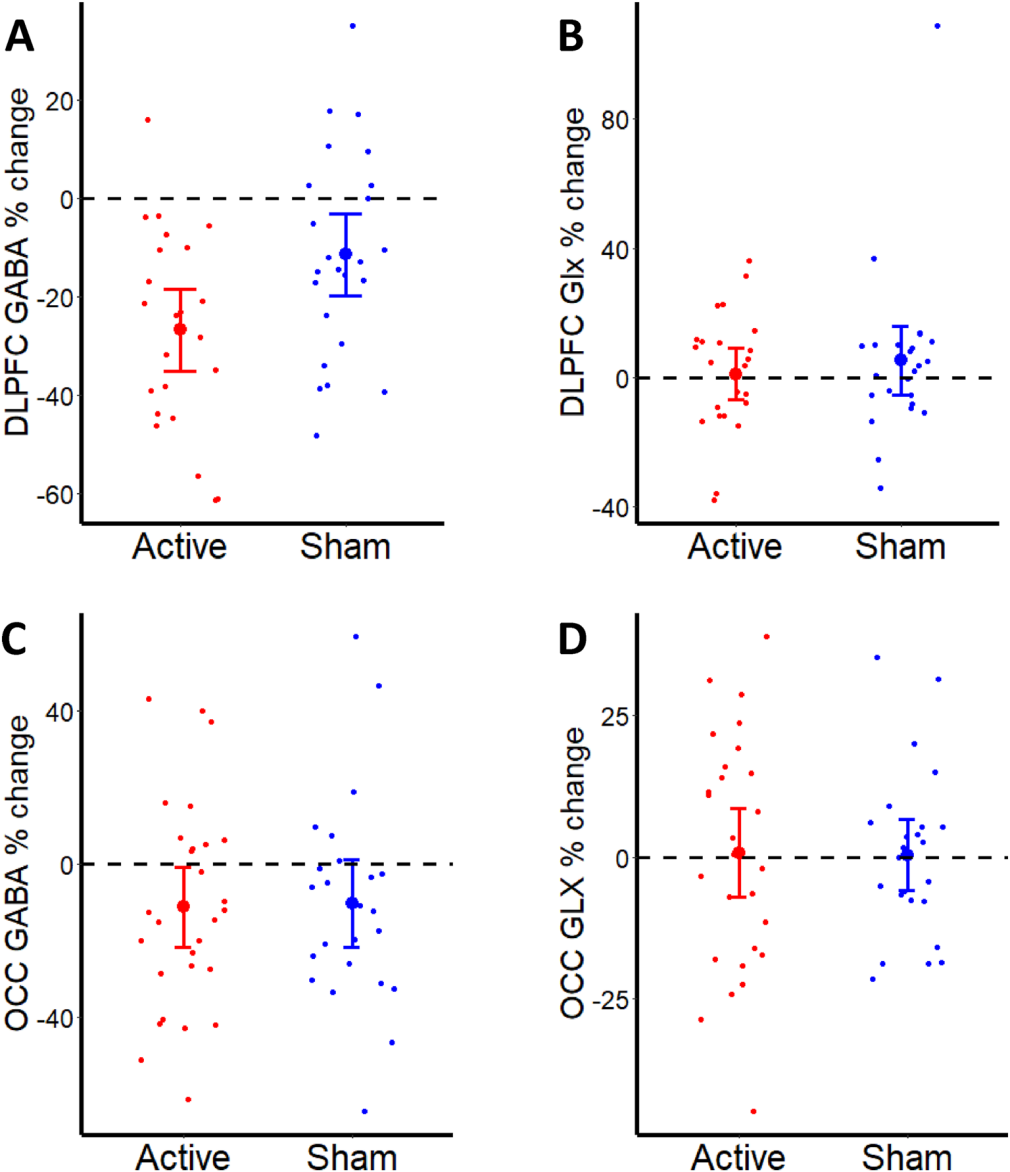
Change (%) in neurotransmitters in the active and sham tDCS groups in (**A**) DLPFC GABA, (**B**) DLPFC GLx, (**C**) Occ GABA and (**D**) Occ Glx. The red and blue bars represent mean ± SEM.

We then wanted to investigate whether atDCS induced the previously reported decrease in GABA+ in DLPFC. AtDCS led to a greater decrease in DLPFC GABA+ relative to sham ($F_{1,46}$ = 4.12, *p* = 0.05, $\eta_{p}^{2}$ = 0.08). There was a 26.77% (SD 4.05%) change in GABA+ in atDCS group as compared to 11.45% (SD 4.07%) change in the sham group. There was no significant effect of atDCS on Glx ($F_{1,46}$ = 0.69, p = 0.41, $\eta_{p}^{2}$ = 0.02). In the control occipital voxel, there was no significant effect of atDCS on GABA + ($F_{1,49}$ = 0.94, p = 0.34, $\eta_{p}^{2}$ = 0.02) or Glx ($F_{1,49}$ = 0.01, p = 0.94, $\eta_{p}^{2}$ = 0.00, Fig. 2).

### Effect of atDCS on BOLD activation measured by fMRI

**Figure 3:**
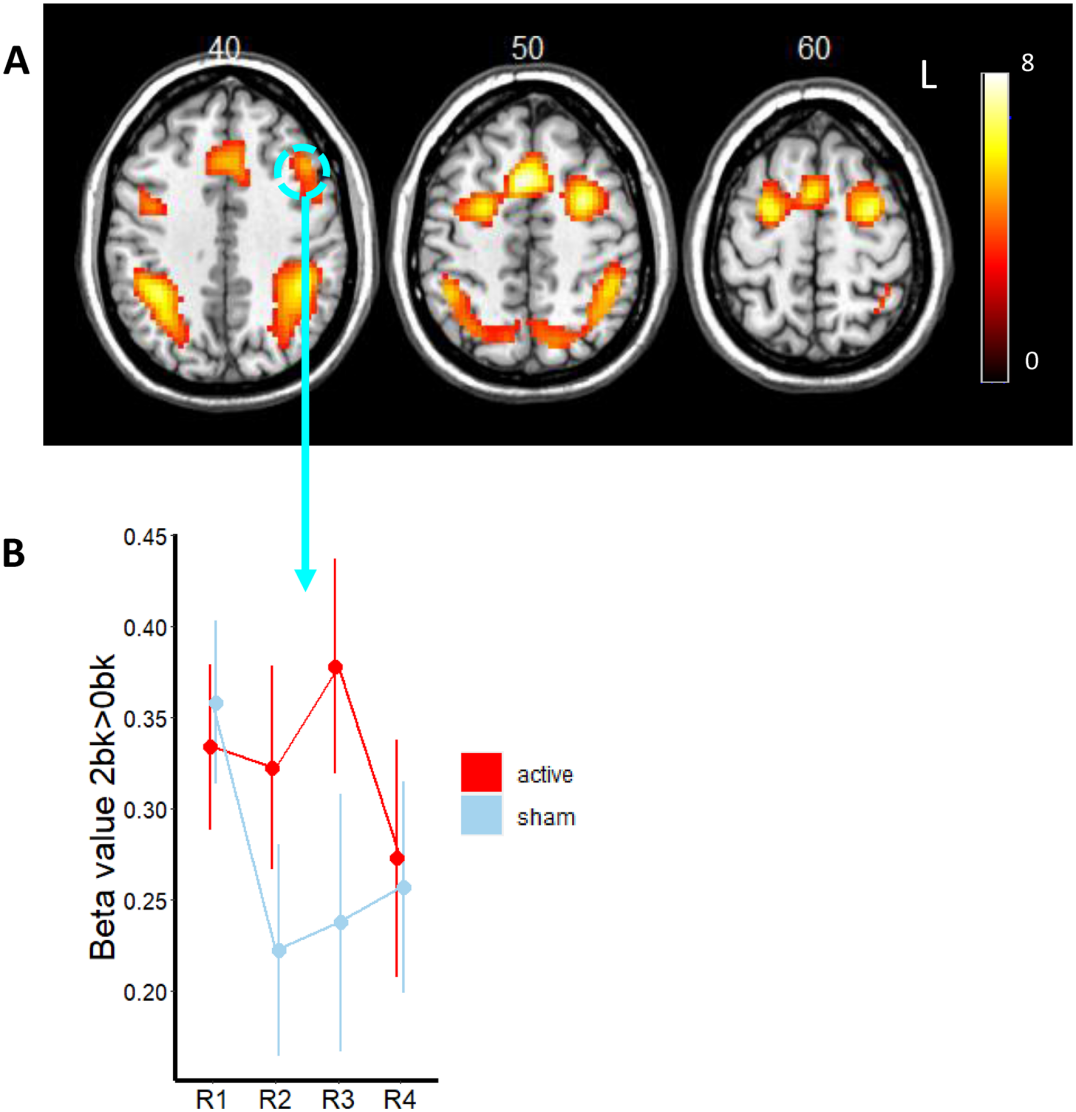
(**A**) Conjunction analysis showing the fronto-parietal activation during the performance. (**B**) Line graph showing the beta values in the left middle frontal gyrus (BA9) across four runs (R1: Run1, R2: Run2, R3:Run3 and R4: Run4). Run1 was acquired pre-stimulation, Runs 2 and 3 were acquired during active or sham stimulation and Run4 was acquired post-stimulation.

During fMRI, participants performed the N-back working memory task (see, the details in the “Methods”). To examine the effects of the task (2back > 0back), we performed a conjunction analysis across the stimulation groups (anodal and sham) at baseline. 2back task relative to 0back induced significant activation in the bilateral prefrontal cortex, intraparietal sulcus, and pre-supplementary motor area (Fig. 3). There was no significant main effect of the stimulation and no interaction between stimulation and session. These results are summarized in Table 2.

**Table 2:** Results of the conjunction analysis at the baseline (2back > 0back).

Region	Cluster size	MNI coordinates	Z score
		$$\begin{array}{*{20}c} x & y & z \\ \end{array}$$	
Middle frontal gyrus	323	$$\begin{array}{*{20}c} {30} & 2 & {53} \\ \end{array}$$	7.22
Pre-supplementary motor area	882	$$\begin{array}{*{20}c} { - 3} & {11} & {50} \\ \end{array}$$	7.05
Superior frontal gyrus		$$\begin{array}{*{20}c} { - 24} & { - 1} & {56} \\ \end{array}$$	6.21
Middle frontal gyrus		$$\begin{array}{*{20}c} { - 45} & {23} & {32} \\ \end{array}$$	5.84
Intraparietal sulcus	1099	$$\begin{array}{*{20}c} { - 36} & { - 46} & {41} \\ \end{array}$$	6.34
Intraparietal sulcus		$$\begin{array}{*{20}c} {42} & { - 43} & {41} \\ \end{array}$$	6.29
Superior parietal lobule		$$\begin{array}{*{20}c} {33} & { - 58} & {44} \\ \end{array}$$	4.95
Middle frontal gyrus	324	$$\begin{array}{*{20}c} {42} & {26} & {35} \\ \end{array}$$	5.77
Frontal pole		$$\begin{array}{*{20}c} {33} & {53} & {14} \\ \end{array}$$	4.11
Precentral gyrus		$$\begin{array}{*{20}c} {51} & 5 & {38} \\ \end{array}$$	3.97

To investigate the effects of the stimulation at the target region during working memory processing, we performed the region of interest (ROI) analysis in the left DLPFC. We used linear mixed models to estimate the effect of stimulation on the level of activation of the DLPFC. Group differences were modelled using fixed effects of predictors (group and fMRI run) and random effect of individual variation. For the ROI analyses in the left DLPFC, there was no significant main effect of stimulation (*b* = − 0.06, se = 0.04, t(71) = − 1.62, p = 0.11) or run (*b* = 0.07, se = 0.12, t(71) = 0.60, p = 0.55) and no interaction between group and run (*b* = − 0.03, se = 0.07, t(76) = − 0.41, p = 0.68). To compare the stimulation effect directly, we performed the planned *t* tests on the DLPFC activation between the groups. We found that aTDCS group maintained task-induced regional activity in the DLPFC during stimulation as compared to sham group during stimulation (t(19) = 1.89, p = 0.05) (Fig. 3). There was no significant difference between groups at the baseline and post stimulation.

### Effect of atDCS of behavioural outcomes

There was no effect of atDCS on Corsi block memory span after accounting for baseline performance ($F_{1,57}$ = 0.84, p = 0.36, $\eta_{p}^{2}$ = 0.01) or Corsi block RT ($F_{1,57}$ = 0.02, p = 0.88, $\eta_{p}^{2}$ = 0.00) or Inverse Efficiency Score (IES) ($F_{1,57}$ = 0.22, p = 0.88, $\eta_{p}^{2}$ = 0.00).

**Figure 4:**
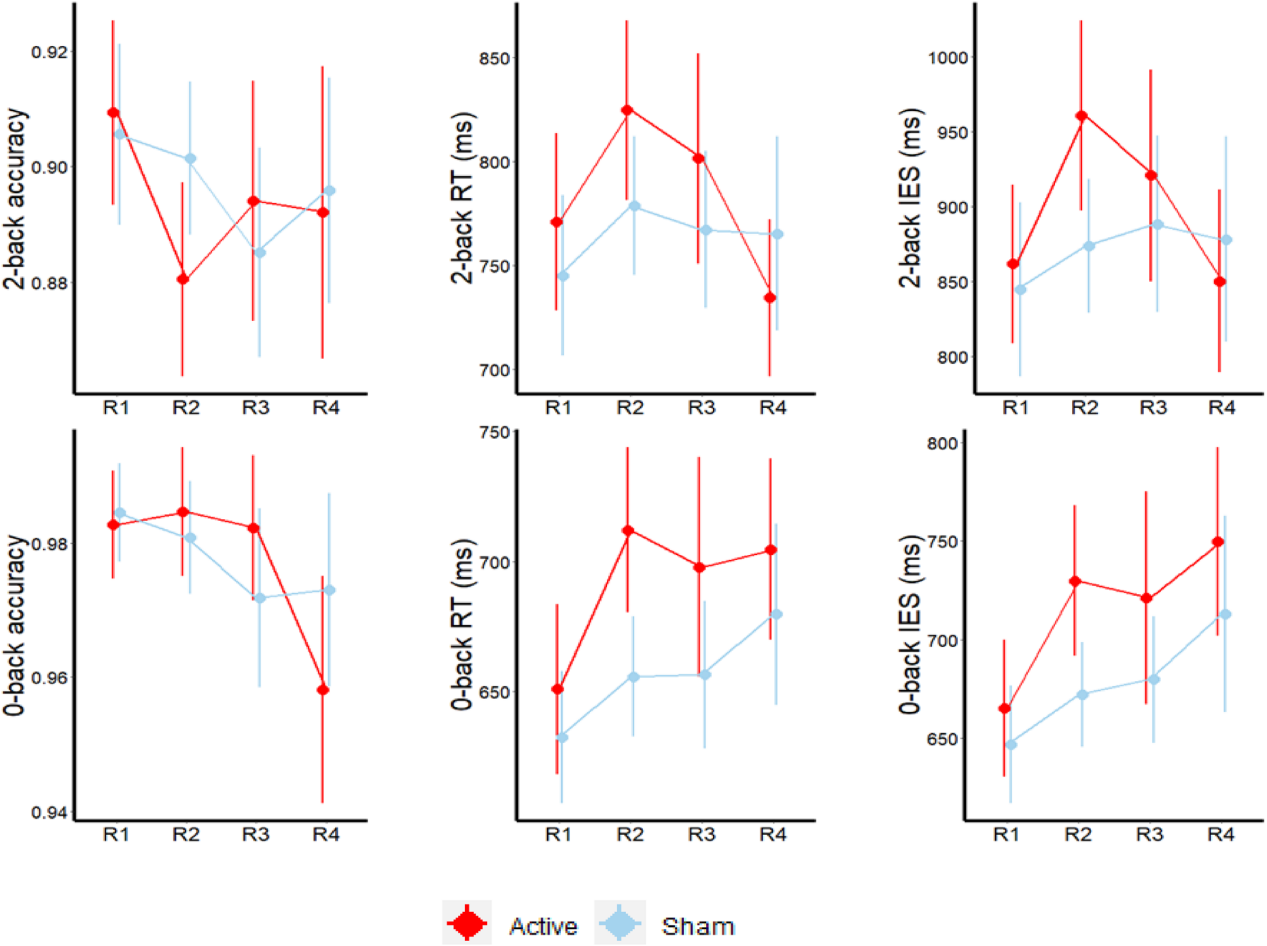
Task accuracy, response time (RT) and Inverse Efficiency Score (IES) on the 2-back and 0-back fMRI tasks across four runs (R1: Run1, R2: Run2, R3:Run3 and R4: Run4). Run1 was acquired pre-stimulation, Runs 2 and 3 were acquired during active or sham stimulation and Run4 was acquired post-stimulation.

On the fMRI behavioural task, we used linear mixed models to estimate the effect of stimulation on the fMRI task accuracy, RT and IES. Similar to ROI analysis presented above, group differences were modelled using fixed effects of predictors (group and run) and random effect of individual variation. On the 2back IES, there was a significant main effect of the group with better speed-accuracy trade-off in the sham group (b = − 47.88, se = 24.18, t(95) = − 1.98, p = 0.05) but no significant effect of run (b = − 23.36, se = 33.45, t(80) = − 0.69, p = 0.48) or any interaction between group and run (b = 34.54, se = 47.22, t(95) = 0.73, p = 0.47). On the 2back task accuracy, there was no significant main effect of group (b = 0.01, se = 0.01, t(95) = 1.13, p = 0.26) or run (b = 0.01, se = 0.01, t(80) = 1.10, p = 0.27) and no interaction of group with run (b = − 0.02, se = 0.01, t(95) = − 1.41, p = 0.16). On 2back RT, there was no significant main effect of group (b = − 30.73, se = 18.12, t(95) = − 1.69, p = 0.09) or run (b = − 9.02, se = 25.07, t(80) = − 0.36, p = 0.720 and no interaction between group and run (b = 4.67, se = 35.41, t(95) = 0.13, p = 0.89) (Fig. 4).

On the 0back IES, there was a significant main effect of the group with better speed-accuracy trade-off in the sham group (b = − 43.06, se = 16.61, t(95) = − 2.59, p = 0.01) but no significant effect of run (b = − 71, se = 22.99, t(80) = − 0.03, p = 0.97) or any interaction between group and run (b = 10.38, se = 32.46, t(95) = 0.31, p = 0.75). On the 0back task accuracy, there was no significant main effect of group (b = 0.00, se = 0.00, t(95) = 0.03, p = 0.97) or run (b = − 0.00, se = 0.01, t(80) = − 0.28, p = 0.78) and no interaction between group and run (b = − 0.00, se = 0.01, t(95) = 0.85, p = 0.40). On the 0back RT, there was a significant main effect of the group with slower response times in the aTDCS group (b = − 40.11, se = 14.41, t(95) = − 2.78, p = 0.01). There was no main effect of run (b = − 4.48, se = 19.95, t(80) = − 0.22, p = 0.82) and no interaction between group and run (b = 9.22, se = 28.18, t(95) = 0.33, p = 0.74).

### Side effects associated with stimulation

We administered a standard side-effect rating scale with rating of 1–3 (none, mild, severe) after each experimental session. All side-effects are listed in Table 3. There were no significant differences in the side effect profile between the aTDCS and sham groups.. There were no mood changes reported by any participants.

**Table 3:** Number of individuals reporting side effects associated with stimulation in both groups.

	Active		Sham		Test statistic
	Frequency of reported symptoms (n)				
	Mild	Severe	Mild	Severe	
Tingling	12	1	5	0	Χ^2^ = 4.03, p = 0.13
Itching	7	1	8	0	Χ^2^ = 1.18, p = 0.55
Warmth	5	2	4	1	Χ^2^ = 0.26, p = 0.88
Burning	1	1	0	0	Χ^2^ = 1.80, p = 0.41
Pain	0	1	3	0	Χ^2^ = 4.41, p = 0.11
Redness	0	0	0	0	
Sleepiness	12	3	9	4	Χ^2^ = 0.43, p = 0.81
Headaches	4	1	3	0	Χ^2^ = 0.95, p = 0.62
Trouble concentrating	11	1	9	1	Χ^2^ = 0.04, p = 0.98
Mood changes	0	0	0	0	

## Discussion

Using a rare genetic disorder known to be associated with GABAergic dysregulation, the goal of this study was to examine how inter-individual differences in GABA relate to working memory and to test the responsiveness of the GABAergic system to the application of atDCS to DLPFC. This is the first such study in adolescents with NF1, showing that atDCS modulates inhibitory activity in the DLPFC. To summarize, we find that higher GABA+ levels were associated with faster response times on the tasks, AtDCS significantly reduces GABA+ and is associated with increased brain activation in the DLPFC as compared to sham stimulation. Surprisingly, we observed poorer task performance during atDCS, but no group differences in task performance post-stimulation.

Our rationale to investigate anodal brain stimulation in the NF1 cohort was based on the findings of the animal data suggesting enhanced GABAergic neurotransmission^25,26^ and human data suggesting hypoactivation of the left DLPFC^23,24^. Our hypothesis was that reduction of GABA and excitatory effect by applying atDCS would improve working memory function. Contrary to our hypothesis, we found that higher baseline GABA+ levels were associated with better task performance; application of atDCS reduced GABA+ and increased activation in the left DLPFC, but this was not associated with improvement in task performance. Indeed, speed accuracy trade-off was poorer in the aTDCS group. Our finding of higher baseline DLPFC GABA+ being associated with better task performance are in line with one previous study in the NF1 cohort, which showed that higher frontal GABA+ was associated with faster responses on inhibitory-control tasks^18^. This is however contrary to findings on studies of healthy populations that suggest that higher frontal GABA is associated with superior cognitive abilities but a more cautious response style^27^. Our findings may therefore indicate a disruption of the relationship between DLPFC GABA and working memory performance and are in line with patterns seen in disorders like schizophrenia^28^.

Performance of a cognitive task and the resultant changes in neural activity may be associated with a variety of neurometabolic effects including changes in GABA, Glx, aspartate, glucose and lactate^29^. Application of atDCS, particularly when combined with a cognitive task has been shown to modulate executive functioning both in healthy subjects and those with underlying psychiatric disorders. We observed significantly greater reduction in DLPFC GABA in response to atDCS as compared to the sham stimulation. AtDCS had no differential modulatory effect on GABA in the occipital cortex showing regional specificity for its effects. We did not see a group difference in Glx in response to left DLPFC stimulation as reported by two previous studies^30^ in healthy populations. This discrepancy may be explained by inclusion in our study of a patient cohort known to be associated with GABAergic dysregulation rather than a healthy population, due to methodological differences such as electrical montage and participants being engaged in a cognitive task whilst the stimulation was being delivered. Our results demonstrate that atDCS modulates neuronal excitability of the DLPFC and merits further investigation in disorders like NF1, where GABAergic overactivity has been shown to underlie cognitive^26^ and social deficits^13^. A significant finding of the study was the magnitude of GABA change in both active and sham groups (up to 60% in both groups) as compared to the more modest changes in Glx. The extent to which tDCS modulates behavior is not associated with a single neurotransmitter, but rather a fine-tuned E/I balance^31^. Future studies using MRS optimized to measure Glutamate (instead of Glx which is Glutamate-Glutamine) in addition to GABA are needed to determine the effect of stimulation on E/I balance and how this relates to learning. Finally, further studies should seek to apply functional MRS techniques to study GABA/Glutamate dynamics during the application of atDCS to better understand neurometabolite responses to neural activation.

The mechanism of action of atDCS is known to be mediated via effects on GABAergic neurotransmission but with possible effects on dopaminergic neurotransmission. Preclinical studies suggest that stimulation leads to a rapid change in glutamic acid decarboxylase (GAD65 and GAD67), resulting in reduced conversion of glutamate to GABA^32^. The prefrontal cortex is heavily regulated by dopamine and stimulation of frontal cortex may lead to an increase in extracellular dopamine concentrations^33^. A recently published study demonstrated that stimulation of left DLPFC in healthy populations is associated with reduction of DLPFC GABA levels but also increase of both GABA and dopamine in the striatum, demonstrating the effect of atDCS beyond the targeted cortical structures^34^. Given the importance of corticostriatal activity in NF1 and other neuropsychiatric conditions, it will be important to determine in future studies whether stimulation of DLPFC has a modulatory effect on the neurotransmitters particularly dopamine in the striatum, given that dopaminergic activity is critically affected in NF1^25,35,36^.

Given the lack of a behavioural response post-stimulation and indeed poorer speed-accuracy trade-off in the aTDCS group, it is important to determine whether application of aTDCS is the right approach for amelioration of working memory impairments in NF1. In healthy populations, several studies suggest that anodal stimulation to the DLPFC can enhance working memory function both in healthy^20^ and psychiatric populations^37^. A recent systematic review of 61 studies involving a single session of atDCS applied to left DLPFC concluded that stimulation enhanced task performance both in speed and accuracy^38^. It is important to note that most of the existing research literature is in adult populations. The brain, particularly the frontal lobe undergoes major remodeling during adolescence shaped critically by the maturation of GABA levels which rise over development^39^. Our results suggest that the atypically developing brain responds differently to tDCS compared to adult brains. It is possible that an alternate approach such as transcranial alternating current stimulation (tACS) may be preferable to tDCS in NF1. Whilst tDCS works by modulating neuronal membrane potential, tACS is thought to operate by modifying endogenous neural oscillations in a frequency-dependent manner^40^. Previous studies suggest atypical neural oscillatory activity in NF1 particularly in the alpha and theta bands^41,42^. Using tACS to directly entrain endogenous oscillations at particular frequency bands may be a fruitful approach. Finally, it is also likely that a single session of tDCS is not enough for a behavioural change^43,44^. It is also possible that behavioural changes develop over a longer period or that repeated sessions of atDCS are required for a measurable impact on behavioural outcomes. The stimulus intensity needs further consideration as more recent studies have shown an inverted U-shaped dose–response curve, in which a moderate stimulus intensity has the strongest effect on cognitive performance^45,46^. Whilst we used 1 mA current based on previous data in paediatric cohorts, alternative (higher or lower) stimulus intensity may need to be considered in the context of an atypically developing brain. Performance fatigue/drowsiness due to a long period of time inside the scanner could also have contributed to the lack of observed behavioural effects.

The fMRI results showed bilateral fronto-parietal activation during the WM task in the NF1 group as previous reported^23^. In the whole brain GLM analysis, there were no differences between the active and sham groups but the ROI analysis showed increased task related activation in the left DLPFC. The increased task related activation during stimulation was however not associated with better task performance. The left DLPFC was chosen given previous reports of hypoactivation in the region during working memory task performance. Our results suggest that it may not be advantageous to enhance brain activity in the region. Given that stimulation parameters including where the electrodes are placed influence stimulation effects, future studies modelling individual current flow should be used to determine ideal tdCS montages^47^. Further work is also needed to investigate whether tDCS is associated with any changes in functional connectivity particularly in the working memory circuitry.

Limitations of our work include use of brief behavioural outcome measures post intervention (chosen to reduce participant burden) and the lack of a healthy control group. It is important to note that GABA levels measured in this study will contain contributions from co-edited macromolecule signal (so-called GABA+), but the relative contribution of these macromolecular signals are thought to be constant and hence unlikely to account for within participant/session GABA changes. Strengths of this study included application of intrascanner atDCS allowing us to probe its effects on GABA function pre- and post-application without moving the participant out of the scanner.

Working memory is of central importance for effective human behavior and an important predictor of academic success^48^. Working memory and other learning impairments are commonly associated with NF1 and other neurodevelopmental and mental health conditions with significant impact on trajectories of academic achievement and overall quality of life. Cognitive remediation techniques may offer some amelioration but there are no effective pharmacological therapies available. Given the strong evidence linking GABA abnormalities to cognitive deficits across neurodevelopmental conditions such as ASD, modulation of GABA using NIBS offers a promising novel therapeutic approach but one that needs to be investigated fully in the context of the developing brain before clinical application. In summary, this is the first study in an adolescent population combining atDCS and MRS online demonstrating direct modulation of neurometabolites with atDCS in real time. Further studies are needed to characterise the neurometabolic effects of atDCS particularly the effects of repeated sessions of atDCS and investigate the use of alternate strategies such as tACS. It will be important to clarify whether there is an effect of NIBS on subcortical structures particularly the striatum, given the important role of cortico-striatal circuitry for complex cognitive functions.

## Methods

### Subjects

Thirty-one adolescents aged 11–17 years were recruited via the Northern UK NF-clinical research network. Inclusion criteria included (i) Clinical diagnosis made using the National Institute of Health diagnostic criteria^49^ and/or molecular diagnosis of NF1; (ii) No history of intracranial pathology other than asymptomatic optic pathway or other asymptomatic and untreated NF1-associated white matter lesion or glioma; (iii) No history of epilepsy or any major mental illness; (iv) No MRI contraindications. Participants on pre-existing medications such as stimulants, melatonin or selective serotonin re-uptake inhibitors were not excluded from participation. The study was conducted in accordance with local ethics committee approval (Ethics reference: 18/NW/0762, ClinicalTrials.gov Identifier: NCT0499142. Registered 5th August 2021; retrospectively registered, https://clinicaltrials.gov/ct2/show/NCT04991428). All methods were carried out in accordance with relevant guidelines and regulations.

### Experimental procedure—cross over intervention design

**Figure 5:**
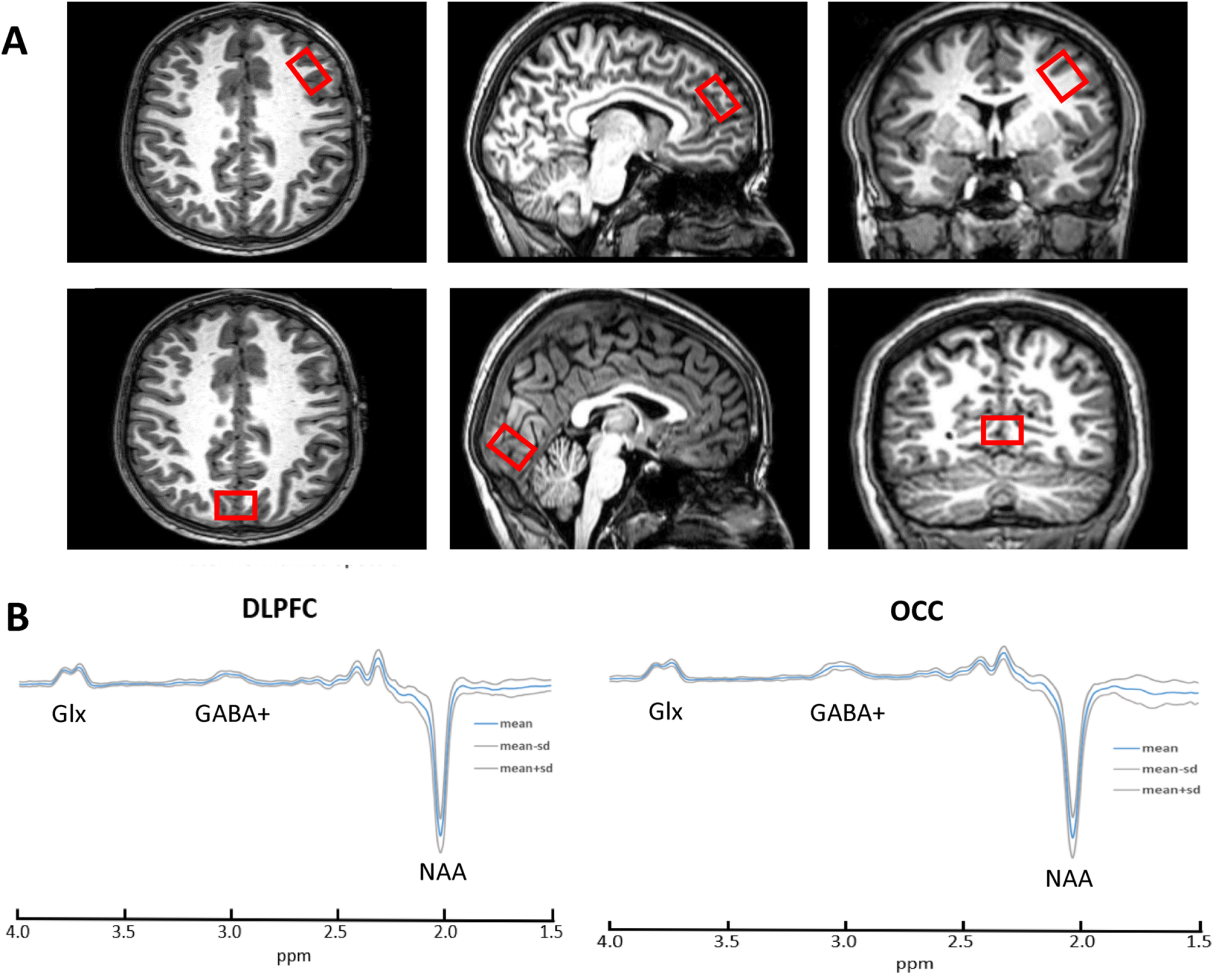
(**A**) Axial, sagittal and coronal images showing the placement of the voxels of interest and (**B**) Composite spectra from Dorsolateral Prefrontal Cortex (DLPFC) and Occipital lobe (OCC). Spectra from individual subjects were frequency aligned and divided by the amplitude of the water signal from each subject's own non-water suppressed acquisition. The spectra were then smoothed with a 3-point running average before the mean and SD. of each individual point across all subjects was calculated. The mean spectrum is shown together with ± 1 SD. The coefficient of variation for the NAA peak was 11% in DLPFC and 15% in OCC.

The effect of atDCS on GABA and working memory was tested using a two parallel-arm, single (participant)-blinded, sham-controlled cross-over design. Each participant had two study visits at least 1 week apart—one with atDCS intervention and with sham as placebo control. No changes were made to the participants medication schedule and did not stop stimulant or any other medication for the research visit. The order of these sessions was randomized and counter-balanced. Baseline assessments (as described below) were conducted at the first visit. Subjects were positioned comfortably in the scanner and a high-resolution T1-weighted image was acquired (see Fig. 5). The T1-weighted image was used to place a voxel of interest (VOI) by hand—over the DLPFC and another VOI in the occipital cortex. After acquiring the MRS from DLPFC and OCC, participants were asked to perform a working memory task for 24 min (4 blocks of 6-min each) during which fMRI data were acquired. AtDCS or sham stimulation was started after the first block of working memory task and continued for 15 min during which the participant engaged in 2 more blocks of working memory tasks. Between each working memory block, participants were asked if they were comfortable and instructions were repeated again. Following tDCS, participants performed the final block of the working memory task. Finally, MRS was acquired again from DLPFC and OCC. T2-weighted images were acquired at the first visit (after the T1 image) and reviewed by a paediatric neuroradiologist (SS) to rule out NF1 associated tumours. The sample size of 31 participants in this study, powered on the expected change of 20% in GABA following tDCS based on our previous work^50^.

### tDCS stimulation

**Figure 6:**
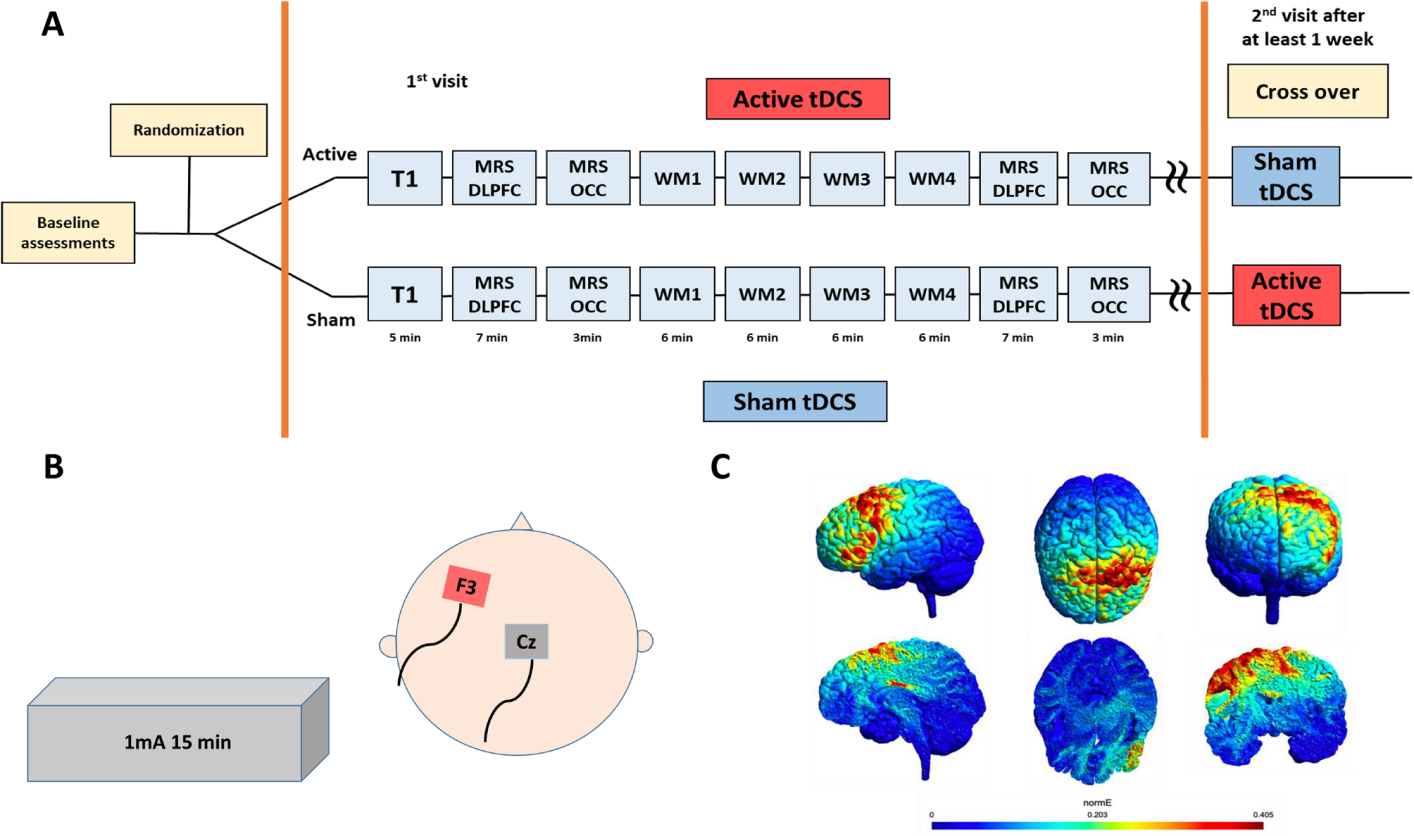
(**A**) Study design demonstrating the imaging sequences acquired in the scanner. WM1-4: Working memory blocks 1, 2, 3, 4. Stimulation started at end of block 1 and continued during blocks 2 and 3. Each imaging sequence was preceded by checking if participant was ok and providing instructions. (**B**) Figure showing the placement of the tDCS electrodes. (**C**) Simulated electric field magnitude (normE, V/m) induced by anodal tDCS.

AtDCS was delivered via a NeuroConn DC-STIMULATOR MR with the anode placed over F3 position in the international 10–20 system and the cathode over the Cz position. Scalp was cleaned with Nuprep gel and Ten20-paste was used as a conductive medium between the scalp and the electrodes. For anodal stimulation, the current was ramped up over 15 s, held at 1 mA for 15 min and then ramped down over 15 s. For sham stimulation, the current was ramped up over 15 s and then immediately turned off. The current parameters were chosen based on our previous experience from a pilot clinical trial of safety in this cohort (clinical trials identifier: NCT03310996). The atDCS induced electrical fields are simulated in Fig. 6. SimNIBS 3.2 (https://simnibs.github.io/simnibs/build/html/index.html) was used to estimate the electric field induced by tDCS^51,52^. The headreco pipeline^53^ was used to segment the different tissue types and create a finite element mesh corresponding to an example T1 image from an open source dataset^54^. The anode and cathode were placed at F3 and Cz respectively, and the standard SimNIBS conductivity values were used.

### Structural and MRS data acquisition and analysis

Scanning was performed on a Philips Achieva 3 T scanner (Best, NL) using a 32-channel head coil. 3D T1-weighed magnetic resonance images were acquired sagittally with a magnetization prepared rapid acquisition gradient-echo sequence (repetition time = 8.4 ms; echo time = 3.77 ms; flip angle = 8°, inversion time = 1150 ms, 0.94 mm in-plane resolution and 150 slices of 1 mm). In addition, a T2-weighted structural scan was acquired (turbo spin echo with TR = 3756 ms, TE = 89 ms, 40 slices of 3 mm thickness and 1 mm gap, in-plane resolution of 0.45 mm). Single voxel ^1^H MRS data were acquired before and after stimulation from two volumes of interest (VOI) in each participant. One VOl (40 × 20 × 24 mm) was placed in the left DLPFC and a control VOI (20 × 50 × 20 mm) was placed within the posterior occipital lobe, centred on the mid-sagittal plane to cover both hemispheres (Fig. 6). Water unsuppressed spectra were acquired from the same locations to act as reference. For detection of GABA+, water-suppressed GABA-edited MEGA-PRESS spectra^55,56^ were acquired with a repetition time of 2000 ms, echo time of 68 ms, 1024 sample points collected at a spectral width of 2 kHz, as previously described^57^. The DLPFC MRS took approximately 7 min to acquire, with 96 averages and OCC voxel took 3 min to acquire with 32 averages. The number of averages were chosen to approximately match spectral quality between DLPFC and OCC.

Quantification was conducted using the Advanced Magnetic Resonance (AMARES)^58^ routine in the Java-based magnetic resonance user’s interface (jMRUI5.1, EU project)^59^. Individual transient frequencies were aligned before constructing the edited spectrum within each acquisition session. To improve the display of the spectra, line broadening of 6 Hz was used. No time-domain filtering was performed on the data before analysis by AMARES. We also rejected MRS data from any subject in which there was a change in NAA line width of greater than 3SD of the global mean linewidth before and after atDCS or sham stimulations such an effect could indicate movement between the two acquisitions. Metabolite resonances including GABA+, glutamate + glutamine (Glx) and *N*-acetylaspartate (NAA) were calculated relative to the unsuppressed water signal from the same voxel. To examine partial volume effects on MRS voxels of interests, the T1-weighted anatomical images were segmented into gray matter (GM), white matter (WM) and cerebrospinal fluid (CSF) using SPM8 (http://www.fil.ion.ucl.ac.uk/spm/). Voxel registration was performed using custom-made scripts developed in MATLAB by Dr. Nia Goulden, which can be accessed at http://biu.bangor.ac.uk/projects.php.en. The scripts generated a mask for voxel location by combining location information from the Philips SPAR file with orientation and location information contained within the T1 image.

In the aTDCS group, 28/29 DLPFC pre-intervention spectra and 24/29 post-intervention spectra were included for analyses (1 pre-intervention and 3 post intervention spectra were rejected due to spectroscopic artefacts such as poor water suppression, lipid contamination or broad line widths and 2 rejected due to > 3SD difference in pre-post intervention NAA line width) and 29/29 pre-intervention OCC spectra and 28/29 post-intervention OCC spectra were included (1 post-intervention spectra not acquired due to technical difficulties). In the sham group, 31/31 DLPFC pre-intervention spectra and 25/31 DLPFC post-intervention spectra were included for analyses (5 rejected due to spectroscopic artefacts and 1 rejected due to > 3SD difference in pre-post intervention NAA line width) and 29/31 pre-intervention OCC spectra and 25/31 post intervention OCC spectra were included for analyses (2 pre and 3 post-intervention spectra not acquired due to technical difficulties, 2 post-intervention spectra rejected due to spectroscopic artefacts and 1 due to > 3SD change in NAA LW). The calculation of partial volume within the VOIs provided the percentage of each tissue type within the relevant voxels. In the aTDCS group, tissue fraction (GM + WM) was 96.9% (± 1.7%) in DLPFC pre-intervention and 96.8% (± 1.7) post-intervention and in the OCC 94.7% (± 2.1%) pre-intervention and 94.7% (± 2.1%) post-intervention. In the sham group, tissue fraction in DLPFC was 97.2% (± 1.8%) pre-intervention and 97.1% (± 1.8) post-intervention, and in the OCC 94.3% (± 2.4%) pre-intervention and 94.3% (± 2.4%). There were no significant differences between the percentage of tissue fraction pre-and post-intervention in any of the voxels. GABA+ was corrected for tissues fraction (GABA/(GM + WM)) for the baseline correlation analyses with behavioural measures of the relationship between GABA and behavioural measures. The MRS quality metrics are presented in Table 4.

**Table 4:** Breakdown of the MRS quality metrics by group and voxel location.

	SNR	Line width (*NAA*)	Line width (*GABA*)
**Active**			
*DLPFC1*	39.44 ± 11.94	5.94 ± 1.65	16.39 ± 2.82
*DLPFC2*	34.23 ± 12.82	7.69 ± 1.87	16.61 ± 2.80
*OCC1*	37.73 ± 12.00	6.47 ± 1.33	16.35 ± 2.53
*OCC2*	34.20 ± 11.49	7.40 ± 1.54	16.45 ± 2.20
**Sham**			
*DLPFC1*	44.33 ± 15.04	5.64 ± 1.39	16.08 ± 3.60
*DLPFC2*	39.82 ± 15.32	6.97 ± 2.01	16.69 ± 2.70
*OCC1*	33.10 ± 14.07	6.08 ± 0.86	16.50 ± 3.19
*OCC2*	34.18 ± 13.44	6.94 ± 1.33	16.08 ± 3.22

### fMRI data acquisition and analyses

Imaging was performed on a 3 T Philips Achieva scanner using a 32-channel head coil with a SENSE factor 2.5. To maximise signal-to-noise (SNR) in the DLPFC, we utilised a dual-echo fMRI protocol developed by Halai et al.^60^. The fMRI sequence included 36 slices, 64 × 64 matrix, field of view (FOV) 224 × 126 × 224 mm, in-plane resolution 2.5 × 2.5 mm, slice thickness 3.5 mm, TR = 2 s, TE = 12 ms and 35 ms. The total volume of each fMRI block was 144.

Image processing and statistical analysis was carried out using SPM12 (Wellcome Department of Imaging Neuroscience, London; http://www.fil.ion.ucl.ac.uk/spm) and MATLAB R2014a. The dual gradient echo images were extracted and averaged using in-house MATLAB code developed by Halai et al.^60^. Functional images were realigned correcting for motion artefacts and different signal acquisition times by shifting the signal measured in each slice relative to the acquisition of the middle slice prior to combining the short and long echo images. The mean functional EPI image was co-registered to the individual T1-weighted image and segmented using the DARTEL (diffeomorphic anatomical registration through an exponentiated lie algebra) toolbox^36^. Then, normalization was performed using DARTEL to warp and reslice images into MNI space and smoothing was applied with an 8 mm full-width half-maximum Gaussian filter. Motion parameters were examined on a participant-by-participant basis. Those demonstrating movement beyond 3 mm translation, or 3 degrees rotation were excluded from the analysis. The fMRI scanning was split into four identical blocks Run1 (baseline), Runs 2 and 3 (during active or sham stimulation) and Run4 (post stimulation). Following pre-processing and removal of data due to motion, the number of participant data available for aTDCS group was 24 in Run1, 23 in Run2, 21 in Run3 and 18 in Run4. In sham group, the number of participant data available was 27 in Run1, 24 in Run2, 21 in Run3 and 23 in Run4.

At an individual level, the bold-oxygen-level-dependent (BOLD) response was modelled using an event-related design where a canonical haemodynamic response function (HRF) was convolved with regressors encoding the onset and duration for the 2 back and 0 back conditions. Additionally, 6 motion parameters were included as regressors in the design matrix. We defined two contrasts of interest (2back > 0 back and 0back > 2 back). In the second level group general linear model, a 2 × 3 ANOVA with stimulation (active vs sham) and sessions (pre intervention, during intervention, post intervention) was conducted for the contrast of interest (2back > 0back) in order to explore the WM related neural patterns and the interaction between group as between subject factor and session as within-subject factor.

To examine the effect of task (2back > 0back) prior to the stimulation, we performed a conjunction analysis across the groups (active and sham). Statistical threshold was set at p < 0.001 at the voxel-level and p < 0.05 at the cluster level with at least 100 contiguous voxels after family-wise error (FWE) correction.

In order to examine the effect of the stimulation on the DLPFC, a region of interest (ROI) analysis was performed using Marsbar^61^. The left DLPFC ROI was defined as a 5 mm sphere in the left middle frontal gyrus BA9 (MNI coordinates: x = − 44, y = 20, z = 32) on a meta-analysis of n-back task in young adults^62^. The beta value of voxel within the ROI was extracted for the 2back > 0back contrast.

### Baseline assessments

Detailed cognitive assessments were carried out to assess working memory at baseline, at the first visit of the participant. Both verbal and visuospatial working memory were assessed using the n-back task. The task was programmed in-house using E-Prime software. Each participant completed verbal and visuospatial tasks at four levels of complexity—0-back, 1-back, 2-back and 3-back tasks. For the verbal task, random letters were presented one at a time and the participant was asked to respond with a key-press if the letter corresponded to the letter one (1-back), two (2-back) or 3 (3-back) letters before. For the 0-back verbal task, participants were asked to press the key to the occurrence of the letter ‘X’. For the visuospatial n-back task, blue squares were presented sequentially on a black 2 × 2 grid. Participants were instructed to respond with a key press if the position of the square matched the position one (1-back), two (2-back) or 3 (3-back) positions before. For the 0-back visuospatial task, participants were asked to respond with a key press to the occurrence of an orange square. The participants were asked to respond as quickly and as accurately as possible to the targets. Each participant was presented with three blocks of each n-back task (24 blocks in total). All stimuli were presented for 500 ms and the inter-stimulus interval was set to 1500 ms. Accuracy was calculated as the proportion of correctly identified hits + correct omissions within each block (correct hits + correct omissions/total responses) averaged across each n-back condition as presented in Table 1. Response times (RT) were calculated only for time to correct response to target stimuli, averaged across each n-back condition. Inverse Efficiency Score was calculated by dividing RT by accuracy (RT/Accuracy) as a measure of speed accuracy trade-off^63^.

Parent-rated Vineland Adaptive Behaviour Scale—third edition^64^ was administered to the parents to assess child adaptive behaviour with overall functioning computed as standardized age equivalent and expressed as an Adaptive Behaviour Composite (ABC). Conners 3 rating scale^65^ was used as a standardized measure for parent reported ADHD symptoms. It consists of 27 items each rated on a 4-point Likert scale (0 = not true at all to 3 = very much true) in five subscales: attention, hyperactivity, learning problems, oppositionality and peer problems. The inattention and hyperactivity subscales are reported below.

### Behavioural outcome measures

At the start and end of each scanning session, while outside the scanner, participants were asked to complete the computerised Corsi block task on the Psychology Experiment Building Language (PEBL)^66^. In this task, 9 identical blue blocks are presented on the screen. These blocks light up on the screen in a sequence, which starts off as a simple sequence of two blocks and increases in complexity based on participant performance. The participant is asked to mimic the sequence observed on the screen. A measure of the memory span and mean RT is reported.

Within the scanner, participants performed 4 runs of working memory tasks during fMRI acquisition—one run each before and after stimulation and two during the atDCS/sham stimulation. Each run consisted of 6 blocks each of 0-back and 2-back verbal working memory task. During the 0-back condition, participants were instructed to press a handheld button when the letter ‘X’ was presented on the screen. For the 2-back condition, the participants were instructed to press the button when the letter on the screen matched the letter 2 screens before. Each block was 30 s long and consisted of 9 target stimuli. Participants were instructed to press a hand-held button when a target was seen, otherwise participants were instructed not to respond. Psychopy2 software was used to display the stimuli and to record responses. Accuracy was calculated separately for 0-back and 2-back tasks (correct hits + correct omissions/total responses). RT were calculated only for time to correct response to target stimuli.

### Statistical analysis

Statistical analyses were performed in SPSS version25 and R version 1.2. Pearson’s correlations were used to investigate the relationship between GABA+ in DLPFC and OCC and the behavioural outcomes. The Fishers Z transformation was used to compare the correlation coefficients. Group differences in MRS metabolites post intervention were analysed using linear regression models adjusting for baseline values of the relevant outcome as a linear covariate. For the fMRI measures, linear mixed modelling was used to estimate the effect group and stimulation on the ROI beta values and for the fMRI task accuracy, RT and IES using the ‘nlme’ package. Overall group differences were modelled using fixed effects of predictors (group and run) and random effect of individual variation. Models were of the general form: *Model*  <- *lme (ROI* ~ run*group, random = *1|ID*). The active group were treated as baseline and parameters were estimated for the sham group. Missing data were handled with the maximum likelihood approach. A p value < 0.05 was considered significant.

### Ethics approval

Ethics approval for the study was obtained from the North West-Greater Manchester South Research Ethics Committee (reference: 18/NW/0762). Written informed consent was obtained from the parents and older adolescent participants and assent was obtained from the younger participants.

## Data Availability

All the datasets included in the project have been deposited on the Sage Bionetworks data repository https://www.synapse.org/. Approved researchers can request to obtain the data which are subject to data sharing agreements.
